# Individual Xenopus Tadpoles Display Distinct and Consistent Visual Preferences

**DOI:** 10.3390/ani16172717

**Published:** 2026-09-01

**Authors:** Cutter J. Barrus, Kaiyuan Zheng, Britney A. Bender, Charlotte A. Fraley, Bradley P. Roth, Sophia M. Glennie, Rowyn P. Birdsley, Kara G. Pratt

**Affiliations:** 1 Department of Zoology & Physiology, University of Wyoming, Laramie, WY 82071, USA; 2 Program in Neuroscience, University of Wyoming, Laramie, WY 82071, USA

**Keywords:** *Xenopus laevis*, vision, behavior, serotonin, visual preference, individuality

## Abstract

Xenopus tadpoles begin displaying several innate visually guided behaviors by developmental stage 48—just 10 days postfertilization. These behaviors include a preference for green over light, and light over dark. Until now, these preferences have been studied by measuring the behavior of groups of tadpoles. The overall behavior of the group, however, does not necessarily reflect the behavior of individuals. Therefore, here we studied visual preferences displayed by individual tadpoles. Our data show that individual tadpoles display varied behavior relative to one another: some tadpoles displayed a strong preference for light over dark while others preferred dark over light; some displayed a strong preference for green over light while others preferred light over green. While preferences varied across tadpoles, preferences displayed within individuals were stable over time (i.e., consistent). Also, previous work on groups of tadpoles shows that visual preferences are shaped by the neuromodulator serotonin (5-HT). At the single tadpole level, we found that inhibiting 5-HT weakened the preference for light displayed by most individuals, and that enhancing endogenous serotonin transmission strengthened the preference for light displayed by most individuals. However, through this, we also identified a small subset of tadpoles that displayed an exceptionally strong preference for light which appeared to be insensitive to experimentally altered levels of 5-HT activity.

## Simple Summary

How neurons self-assemble into circuits that give rise to behaviors is a fundamental question in neuroscience. We address this question in the visual system of the *Xenopus laevis* tadpole. Xenopus tadpoles begin displaying several innate visually guided behaviors by developmental stage 48—just 10 days postfertilization. These behaviors include a preference for green over light, and light over dark. Until now, these studies have been carried out using groups of tadpoles. The behavior of the group, however, does not reflect the behavior of individuals. Therefore, here we studied preferences displayed by individuals. We found that while preference strengths varied across individuals, they were consistent within individuals, suggesting behavioral individuality. Overall, this work forms the basis for exploring how specific visual preferences are manifested at the visual system (circuit) level.

## 1. Introduction

During their juvenile or larval stages of development, many visual species of animals display innate preferences for specific colors and/or light intensities. These innate preferences have been studied across a wide range of animals including newly hatched turtles [1,2], tadpole and zebrafish larvae [3,4,5,6], crayfish [7,8], and drosophila [9,10]. These innate behaviors are thought to promote survival by guiding the young, who are especially vulnerable to predators at this time in their lives and who have not had time for experience-dependent learning, to sources of food and safety, and away from danger.

The focus of this study is on the innate visual preferences displayed by the *Xenopus laevis* tadpole. There are many features of this amphibian that render them especially suitable for this study. For one, this amphibian’s visual system serves as a model to study how neural circuits form and give rise to behaviors. Hence, there is a broad base of knowledge on the development of the circuits that comprise their visual system, and well-established approaches for linking circuit function with visually guided behaviors [11,12,13]. Every stage of development, from zygote to adult, has been identified and labeled [14]. In addition to these experimental advantages, there are several aspects of *X. laevis* that render them particularly interesting from the evolutionary and ecological standpoints: first, this species is incredibly evolutionarily conserved, evolving from the hybridization of two unknown progenitor species approximately 17 million years ago [15]. It is noteworthy that they have not changed since then. Second, (which may be linked to the first) they are infamously invasive, and have a high tolerance for anoxic conditions, drought, and can starve for up to twelve months [16]. Hence, studying the behaviors and preferences of the *X. laevis* tadpole provides a glimpse into this frog’s extraordinary success.

Our previous work on visual preferences showed that groups of tadpoles display a moderate yet persistent preference for the color green over light [4], and a slightly stronger and equally persistent preference for light over dark [17]. Thus, for groups of tadpoles, the overall hierarchy of preference strengths was determined to be green > light > dark. One reason why tadpoles may display a preference for green is because green is the color of the aquatic plants that serve as their food and shelter. Consistent with this theory, a field study determined that tadpoles spend a majority of their time residing near green aquatic plants, but not when the aquatic plants had experimentally been bleached white [18]. However, we also observed that this preference for the color green is not especially strong, but moderate. If the color green truly represents the location of food and shelter, why is the preference for this stimulus only moderate in strength? This apparent discrepancy may be generated by two opposing visually guided behaviors—both of which promote survival: (1) the drive to reside in regions of safety and food, and (2) the drive to not be identified by predators. The shell game theory of predator–prey interactions predicts that animals (especially prey) must avoid being predictable in order to evade predators [19]. This led us to ask how this moderate—perhaps ecologically optimal—preference may be achieved by the group.

To determine this we measured strength of preference for light vs. dark and green vs. light displayed by individual tadpoles who had been reared under nearly identical conditions. For both tests, we observed a significant degree of variability in preference strength across individuals, but consistent preferences within individuals. The data also suggest that differential levels of endogenous serotonin may underlie the variability in preference strengths displayed by the majority of individuals.

## 2. Materials and Methods

### 2.1. Animal Husbandry

**Figure 1:**
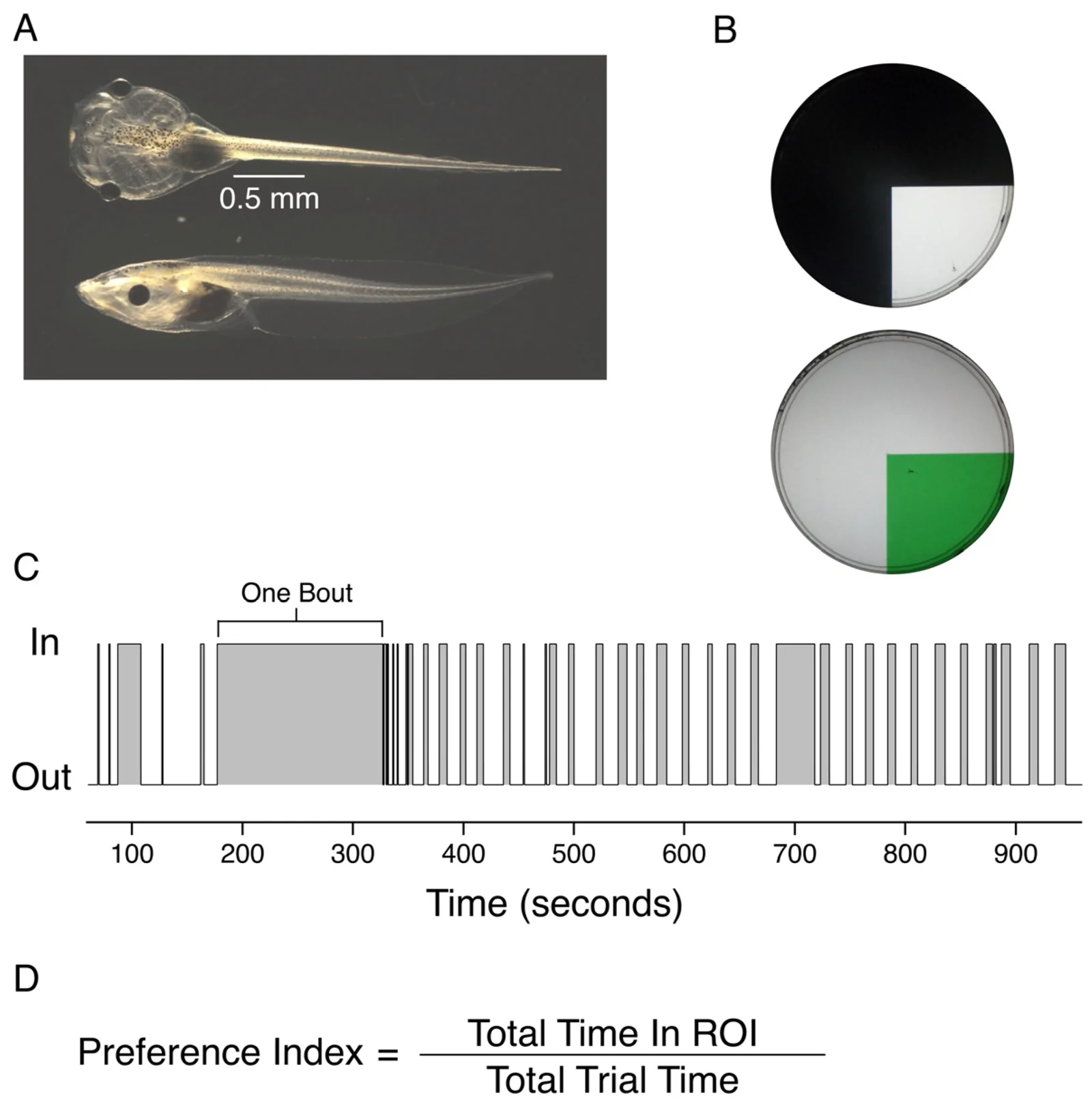
Test for measuring long-term visual preferences displayed by freely swimming individual *X. laevis* tadpoles. (**A**) Overhead and sagittal views of a 12-day-old (developmental stage 48) *Xenopus laevis* tadpole. (**B**) Schematic of the test dish utilized to measure (top) the preference for light versus dark and (bottom) the preference for green versus light. The region of interest (ROI) is 1/4th the dish while the remainder is what is being pitted against the ROI. (**C**) Example of the bout plots showing when the tadpole is in the ROI and when the tadpole is out of the ROI. (**D**) The mathematical equation used to obtain the preference index scores for each individual tadpole.

All animal husbandry and experimental procedures were approved by University of Wyoming’s Institutional Animal Care and Use Committee (IACUC; protocol #2023-0066, approval date 19 September 2023). All tadpoles were obtained through in-house mating of adult wild-type *Xenopus laevis* frogs. Tadpoles were raised in Steinberg’s solution and housed on a 12:12 light/dark cycle in white-walled incubators at 22 °C. Visual preferences were measured across developmental stages 48 and 49 (approximately 11–20 days postfertilization, dpf). Figure 1A shows an image of a development stage 48 *X. laevis* tadpole. Developmental stages are in accordance with Nieuwkoop and Faber [14]. Each test group comprised no fewer than 3 clutches.

### 2.2. Open-Field Individual Visual Preference Assay

A slightly modified version of the experimental paradigm described in Hunt et al. [4] was used to quantify an individual tadpole’s visual preference. This paradigm utilizes a 14 cm diameter circular Petri dish filled with 75 mls of Steinberg’s to create a depth of 6 mm. To minimize any extraneous visualize stimuli, the outside wall of the dish was surrounded by black construction paper, and the trials were carried out in an un-lit room. The dish was positioned atop an LCD computer monitor which was projecting a circular stimulus which comprised one quadrant (25% of the area of the test dish), referred to as the region of interest (ROI), and the remaining 3 quadrants (75% of the surface area of the dish) displaying the opposing stimulus. For testing the preference for light vs. dark, the ROI (quadrant) was light, and the remaining 3/4 s of the dish was dark (Figure 1B). To test the preference for green vs. light, the ROI quadrant was green and the remainder of the dish, light (Figure 1B). For both of these preference tests, the visual stimuli were static, and the quadrant ROI appeared in the same position in the test dish—the southwest quadrant—for all trials. Visual stimuli were created using Adobe Photoshop Creative Cloud 2018, Lab color mode. The luminosities and spectral composition were identical to those used in our previous behavioral studies [4,17]. The white light stimulus had a Lightness Channel (L) value of 100. The dark stimulus had an L value of 0. The green stimulus had Lab values of L: 75, a: −75, b: 75 (closest RGB: 0, 216, 0). All stimuli were displayed on a computer monitor and were illuminated from behind (i.e., back-lit); thus, the light, dark, and green stimulus all include some level of light in addition to their luminance levels assigned in Photoshop. The different stimuli do, however, retain their relative differences in luminosity when displayed on a back-lit monitor, e.g., the displayed white light stimulus is brighter than the dark stimulus, and the green light stimulus is darker than the white light stimulus (L 75 vs. L 100, respectively). For simplicity, throughout this report the white light stimulus will be referred to as “light”, the relatively darker stimulus as “dark”, and the green light stimulus as “green”. One wild-type Xenopus tadpole was transferred to the dish and allowed to acclimate for 1 min. Their behavior was recorded for 15 min with a digital video camera with a resolution of 60 frames per second (GoPro, San Mateo, CA, USA). All experiments were carried out between 12:00 noon and 5:00 pm to avoid any circadian-dependent differences [17]. Tadpoles in this study were randomly chosen. The criteria for being chosen was the display of normal developmental stage 48 swimming patterns, characterized by essentially constant swimming and a head-down, tail-up body posture. The sex of the individuals was not determined. Group sample size (between 10 and 25 individuals per group) was based on previous work which measured visual avoidance displayed by individual Xenopus tadpoles [20].

### 2.3. For Measuring Consistency of Preferences Displayed by Individuals

After the 15 min trial, the individual tadpole was transferred to a bowl of Steinberg’s solution, singly housed for identification purposes. To measure consistency of preferences, tadpoles were re-tested the next day (Day 2) on the same test that they took on Day 1 (either light versus dark or green versus light).

### 2.4. For Measuring the Effect of Pharmacologically Altered Serotonin (5-HT) Activity Levels on Preferences Displayed by Individuals

**Figure 2:**
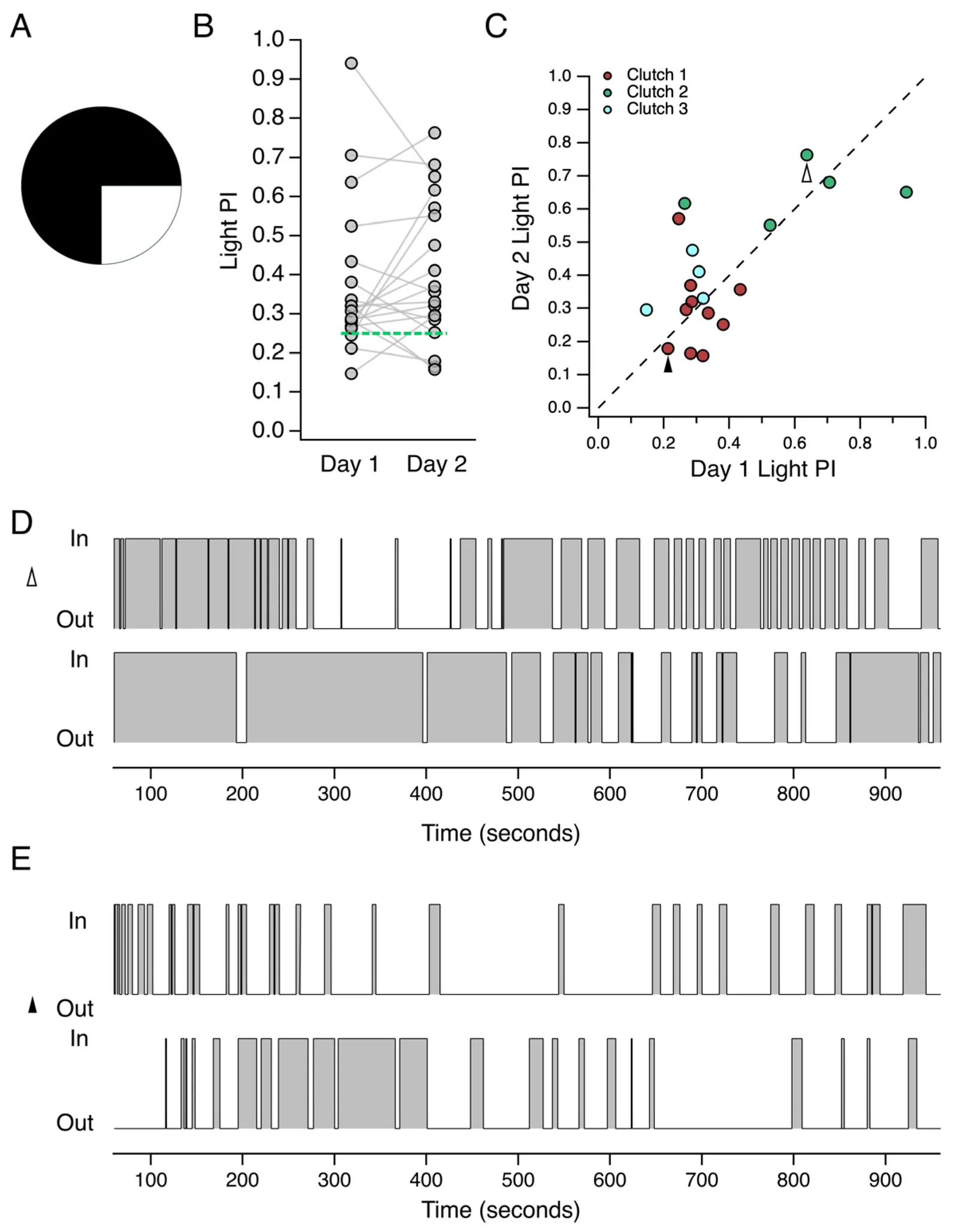
Light vs. dark experiments indicate that individual tadpoles display an innate, disperse, and enduring preference or avoidance for light. (**A**) Light vs. dark stimulus: 1/4th light and 3/4th dark. (**B**) Graph showing the light preference index of the individual tadpoles across Day 1 and Day 2 tests. Each dot and line corresponds to an individual tadpole’s preference index on Day 1 and 2 (*n* = 19). The green dashed line at the preference index of 0.25 represents indifference (i.e., no preference or avoidance, aka neutral). Coefficient of variation (CV) values indicate significant variability across the group of individuals (CV on Day 1: 51.8%, Day 2: 45.3%). (**C**) The same data from panel B graphed with preference for light on Day 1 (x-axis) and preference index for light on Day 2 (y-axis). Each dot corresponds to an individual tadpole’s preference on Day 1 and Day 2 (ICC: 0.651, 95% CIs: 0.295, 0.849). The dashed line is the identity line. The highlighted tadpole’s swimming patterns were displayed in panels (**D**,**E**). (**D**) Bout plots showing each time that tadpole 41 entered and exited the light region of the dish throughout the 15 min (i.e., 60–960 s) trial. The preference index scores are similar (0.64 and 0.76, respectively). (**E**) Bout plots showing each time that tadpole 38 entered and exited the light region of the dish throughout the 15 min (i.e., 60–960 s) trial on Day 1 (top) and then Day 2 (bottom). The preference index scores are similar (0.21 and 0.18, respectively).

To measure the effect of altering serotonin activity levels on individual preferences for light, individual tadpoles were tested as usual on Day 1 on the light versus dark test (Figure 2A) to obtain a baseline preference score. Immediately after testing, the individual tadpole was transferred to a bowl (again, singly housed) that contained either 5 uM of pCPA (tryptophan hydroxylase inhibitor which reduces 5-HT levels), or 1 uM fluoxetine (SSRI to enhance 5-HT activity). Individuals were then re-tested on Day 2 (approximately 24 h later) to measure their post-pCPA or post-fluoxetine preference strengths.

### 2.5. Analysis of Preference Test Videos

To quantify preferences of individual tadpoles, the total amount of time the tadpole spent in the ROI was computed by summing all the individual bout durations (the time at which the tadpole exited the ROI—the time at which the tadpole entered the ROI; Figure 1C). Analysis was carried out manually, by human eye. A tadpole was considered in the ROI if at least one of its eyes and a majority of its head was in the region [4]. While the primary analyzer was not blind to condition, many randomly selected trials were re-analyzed by a different researcher who was blind to condition. The results of the re-analysis were essentially identical to that of the initial one, underscoring the objective nature of the analysis.

The preference index was calculated by dividing the total amount of time spent in the ROI by the total trial time (15 min) (Figure 1D).

### 2.6. Pharmacological Agents

To pharmacologically decrease serotonin levels, tadpoles were exposed to the tryptophan hydroxylase inhibitor 4-Chloro-DL-phenylalanine methyl ester hydrochloride (pCPA; 5 uM; Sigma-Aldrich, St. Louis, MO, USA, catalog C3635-1G). To enhance serotonin transmission, the tadpoles were exposed to the selective serotonin reuptake inhibitor (SSRI) N-methyl-3-phenyl-3-[4-(trifluoromethyl)phenoxy]propan-1-amine (fluoxetine, 1 uM; Sigma-Aldrich, catalog #F132). The 1 uM solution of fluoxetine was prepared from a 10 mM stock dissolved in 100% dimethyl sulfoxide (DMSO); thus, the final “working” solution (1 μM fluoxetine) also contained 0.01% DMSO, a trace amount that has been previously determined not to interfere with tadpole visual preferences including the fluoxetine-induced strengthening of the preference for light, their normal swimming pattern (stereotypical heads-down, tails-up body posture and tails constantly fluttering), or on the functioning of their visual circuitry [17,21,22]. The concentrations of pCPA (5 μM) and fluoxetine (1 μM) were used in accordance with previous work that shows the drug does not alter the normal heads-down, tails-up swimming pattern [17]. Using number of swimming bouts as a proxy for locomotor activity and plotting this versus the preference score indicates no obvious relationship between the number of times the tadpole moved into and out of the quadrant and their overall preference score. For control- and drug- treated tadpoles we observed, for example, very low number of bouts associated with both weak and strong preferences, and high number of bouts associated with both weak and strong preferences. In all experiments utilizing pharmacological agents, the appropriate drug was applied 24 h before behavioral assessment.

### 2.7. Statistical Analysis

All descriptive statistics are reported as coefficients of variation (CV) and standard deviation. The Shapiro–Wilk test was used to determine whether or not a set of data followed a normal distribution. These tests required non-parametric statistical analyses (light vs. dark Day 1 vs. Day 2, respectively: *W* = 0.802, *p* = 0.0012, *W* = 0.936, *p* = 0.2264, *n* = 19; green vs. light Day 1 vs. Day 2: *W* = 0.867, *p* = 0.006, *W* = 0.903, *p* = 0.03, *n* = 23; pCPA light vs. dark Day 1 vs. Day 2: *W* = 0.878, *p* = 0.082, *W* = 0.793, *p* = 0.008, *n* = 12). A Wilcoxon matched pairs signed-rank test was carried out for non-normal data. If the data are normal, a two-tailed paired *t*-test was utilized. Test–retest reliability of the preference index from Day 1 to Day 2 in the same tadpoles was assessed using a two-way absolute-agreement single-measure intraclass correlation coefficient (ICC). We generated 95% confidence intervals (CIs) for repeatability estimates. It was determined, using the interquartile range (IQR) and the five-number summary, that there were two statistical outliers in the green vs. light test. Therefore, those two points were left out in statistical analysis. Statistical analyses were carried out using R (2026.06.0, Build 242), Excel (Ver. 2605, Build 20026.20182), and GraphPad Prism (2026.05.11, Ver. 11.0.2 (92)).

## 3. Results

### 3.1. Individual Tadpoles Display Distinct and Consistent Visual Preferences for Light over Dark and Green over Light

Results of the light versus dark test (Figure 2A) show that while most tadpoles preferred to be in the light quadrant, the strength of this preference varied significantly across individuals (Figure 2B,C; Table 1). Preference index scores ranged from 0.15 to 0.94. To determine whether these individual preferences were stable over time, the same tadpoles were re-tested the next day (approximately 24 h later). We found that individuals displayed similar preferences on Day 2 (Table 1). In other words, an individual who displayed a relatively strong preference for light on Day 1 displayed a similarly strong preference on Day 2 (Figure 2B,C). Figure 2D and E show swimming bout plots (location of tadpole in test dish across the 15 min trial) for two individuals who displayed especially different, yet, within each individual, especially consistent preferences for light over dark. Color coding individuals based on kinship reveals a course, clustered pattern that suggests there may be less variability within tadpoles of the same clutch compared to inter-clutch variability (Figure 2C), although inter-clutch variability was determined to be statistically nonsignificant. Overall, we observed significant variability in the strength of the preference for light across individuals, but stable preferences within individuals.

**Figure 3:**
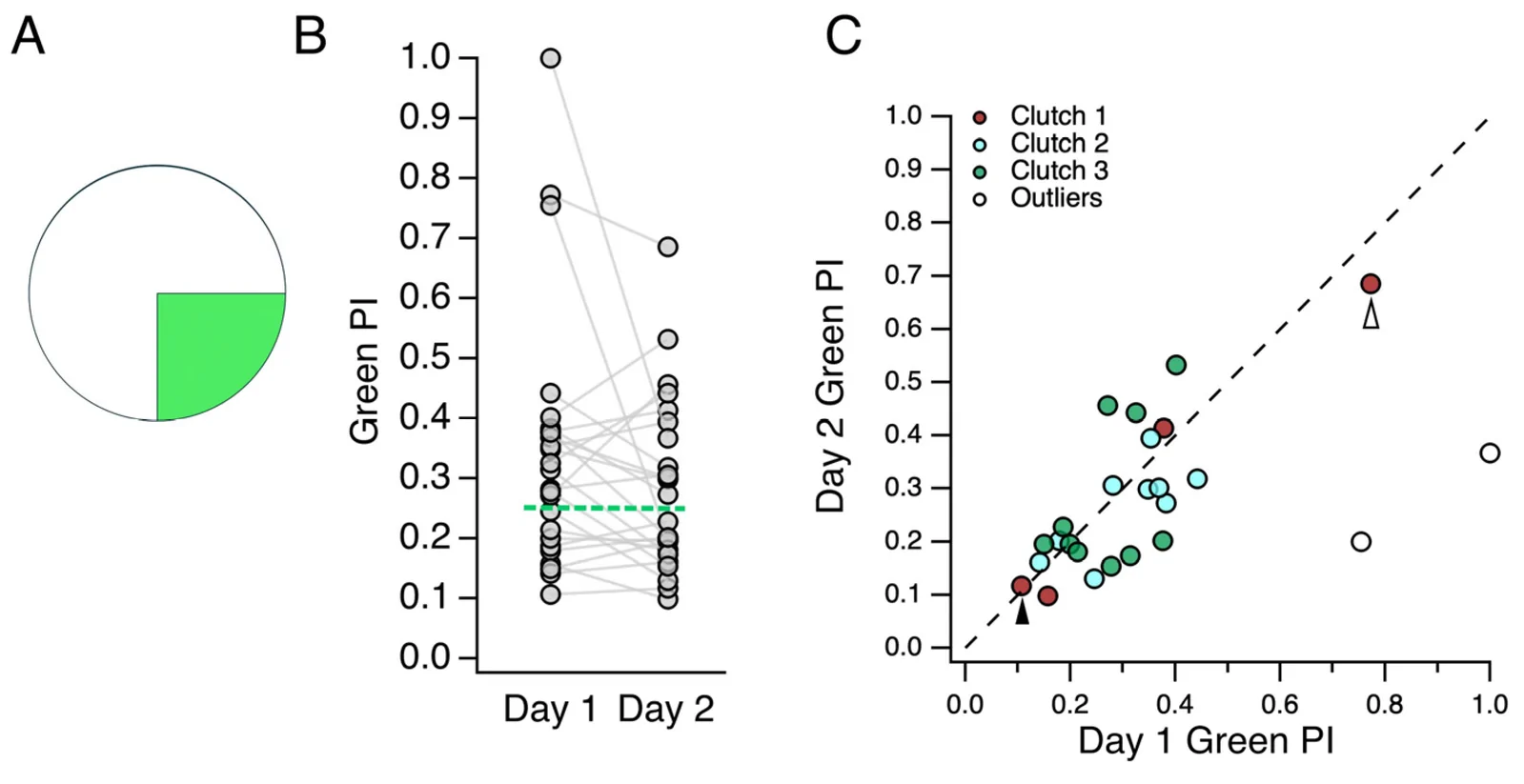

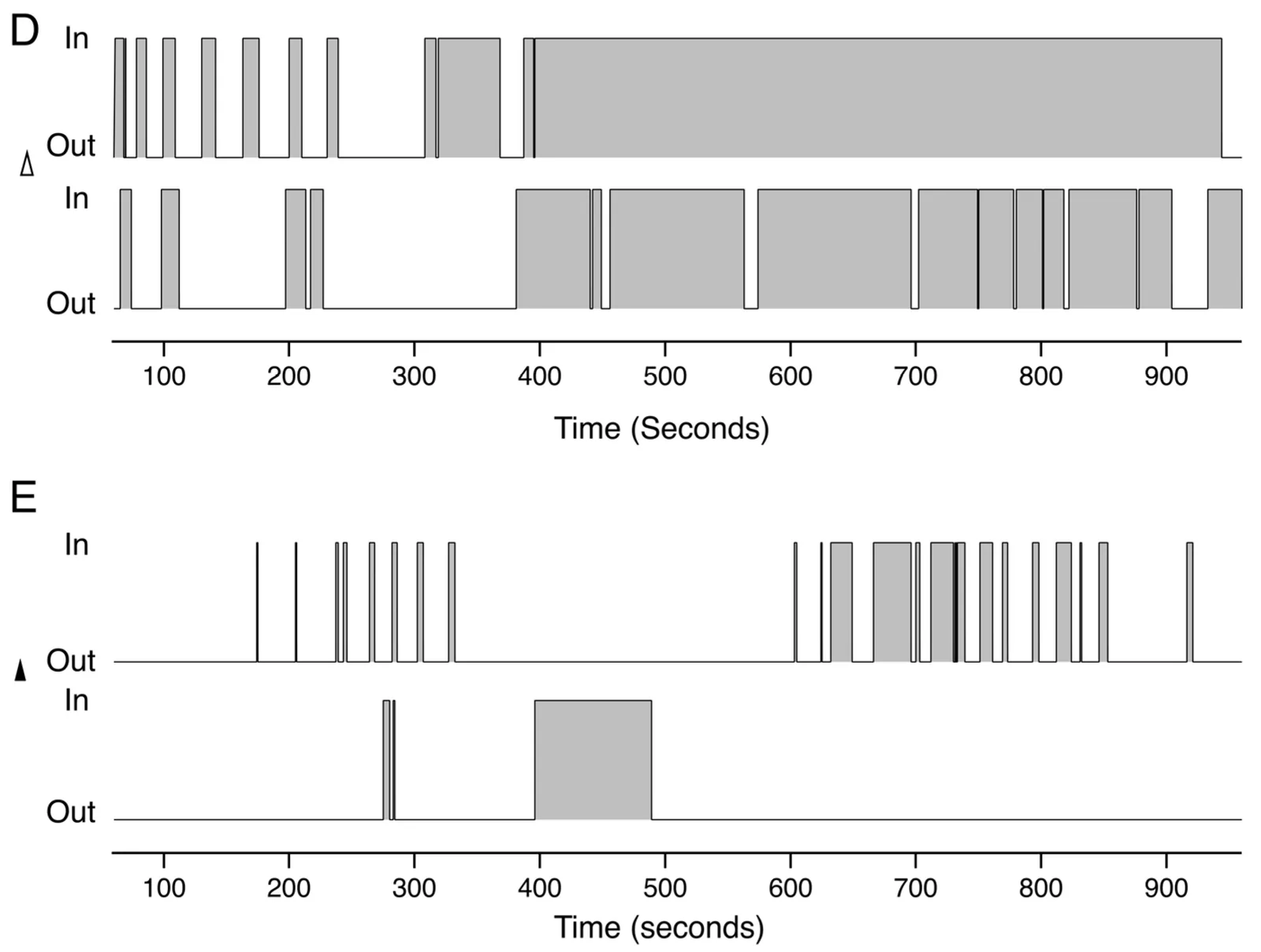
Green vs. light experiments indicate that individual tadpoles display an innate, distinct, and enduring preference or avoidance for green. (**A**) Green vs. light stimulus: 1/4th green and 3/4th light. (**B**) Graph showing the green preference index of the individual tadpoles across Day 1 and Day 2 tests. Each dot and line corresponds to an individual tadpole’s preference index on Day 1 and 2 (*n* = 23). The green dashed line at the preference index of 0.25 represents indifference (i.e., no preference or avoidance, aka neutral). Coefficient of variation (CV) values indicate significant variability across the group of individuals (CV on Day 1: 47.3%, Day 2: 52.7%). (**C**) The same data from panel B graphed with preference for green on Day 1 (x-axis) and preference index for green on Day 2 (y-axis) to clearly illustrate the similarities between Day 1 and Day 2 (ICC: 0.717, 95% CIs: 0.445, 0.869). Each dot corresponds to an individual tadpole’s preference on Day 1 and Day 2. The dashed line is the identity line. The highlighted tadpole’s swimming patterns were displayed in panels (**D**,**E**). (**D**) Bout plots showing each time that tadpole 3 entered and exited the green region of the dish throughout the 15 min (i.e., 60–960 s) trial. The preference index scores are similar (0.77 and 0.69, respectively). (**E**) Bout plots showing each time that tadpole 4 entered and exited the green region of the dish throughout the 15 min (i.e., 60–960 s) trial on Day 1 (top) and then Day 2 (bottom). The preference index scores are similar (0.11 and 0.12, respectively).

Based on our previous work showing that groups of tadpoles display a moderate yet persistent preference for the color green over light [4], we next measured the strength of this preference displayed by a different set of tadpoles to determine how the preference of the group is manifested (Figure 3A). While the data show that the majority of tadpoles displayed a preference for green over light, once again we observed a significant degree of variability in preference strength (Figure 3B,C). Preference index scores for green over light spanned from 0.11 to 1.0 (Figure 3B,C). Re-testing individuals on the same green versus light test the next day indicated that the specific strength of preference for green is maintained for at least 24 h (Figure 3B,C; Table 1). In contrast to the clutch-dependent pattern observed for the preference for light over dark, intra-clutch variability appeared similar to that displayed by individuals across clutches. This suggests that the specific preference for green over light may not involve a heritable factor. Figure 3D,E show swimming bout plots of two individuals who displayed markedly different preferences for green over light between them, but consistent preference within them.

Overall, these results indicate a significant amount of variability in the strength of the preference for green across individuals while the strength of preference displayed within individuals appeared relatively stable.

### 3.2. Pharmacologically Altering Levels of Serotonin Transmission Modulates the Strength of the Preference for Light over Dark Displayed by Most, but Not All, Individuals

**Figure 4:**
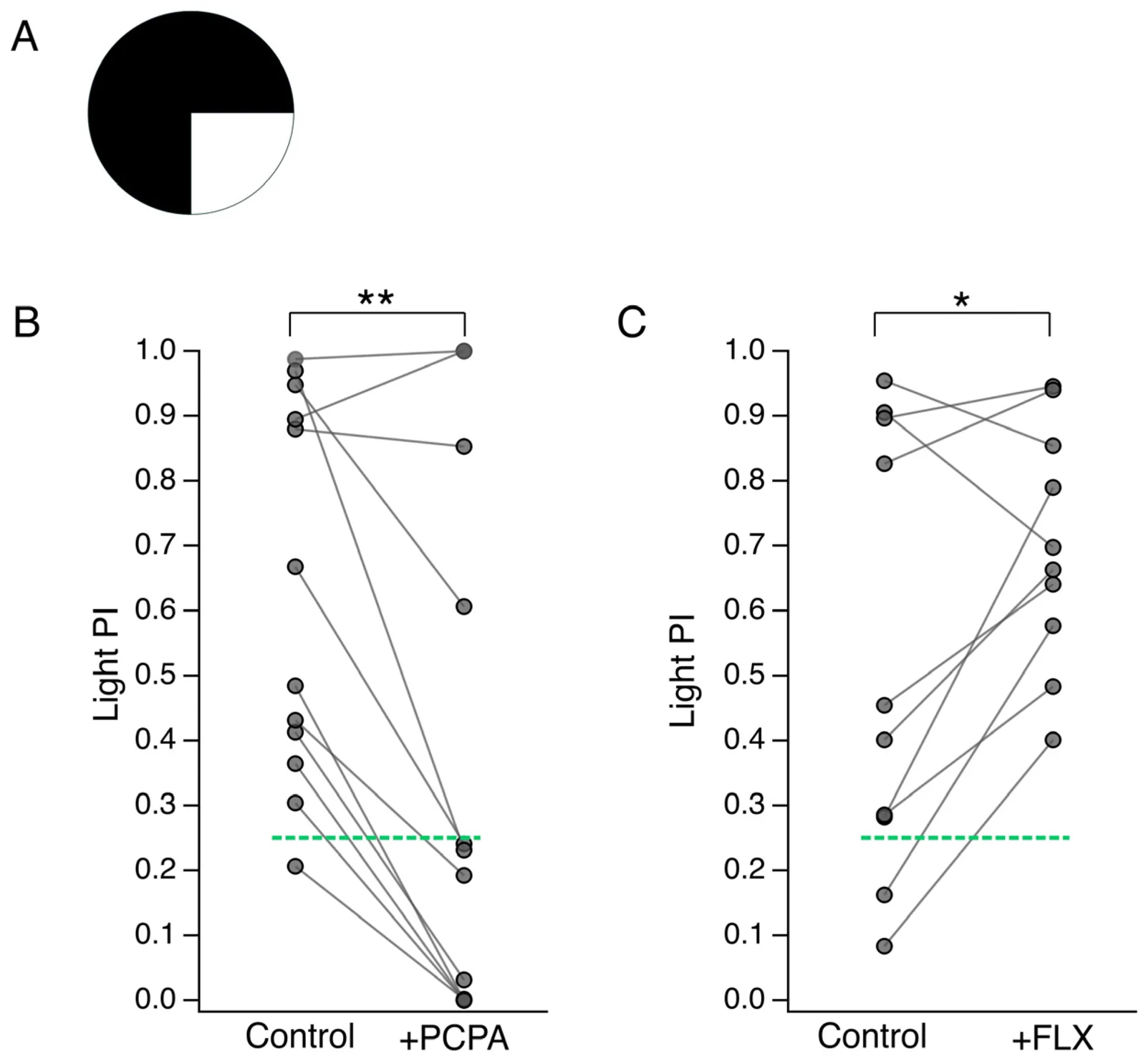
Pharmacologically altering 5-HT activity levels modulated the preference for light over dark displayed by the majority of individuals. (**A**) Light vs. dark stimulus: 1/4th light and 3/4th dark. Graph showing preference index values for individual tadpoles (**B**) before (Control) and after 24 h exposure to pCPA. The 24 h exposure to pCPA significantly decreased the average preference for light (*p* = 0.003; *n* = 12). Note the few that displayed a strong preference for light both before and after pCPA. Graph showing preference index values for individual tadpoles (**C**) before (Control) and after 24 h exposure to fluoxetine. The 24 h exposure to fluoxetine (FLX) significantly increased the average preference for light (*p* = 0.033; *n* = 10). Note that, similar to (**B**), there are a few individuals that displayed a strong preference for light both before and after fluoxetine. In both (**A**,**B**), each dot and line corresponds to an individual tadpole’s preference index before and after drug exposure, and the green dashed line at the preference index of 0.25 represents indifference. * *p* < 0.05; ** *p* < 0.01.

Our previous work implicates a role for the neurotransmitter serotonin (5-HT) in shaping the strength of the preference for light displayed by tadpoles [4,17]. Specifically, we found that experimentally enhancing 5-HT transmission by exposing tadpoles for 24 h to the selective 5-HT reuptake inhibitor (SSRI) fluoxetine significantly strengthened the preference for light displayed by groups of tadpoles, and that, conversely, experimentally inhibiting 5-HT levels by exposing tadpoles for 24 h to the tryptophan hydroxylase inhibitor, pCPA, decreased the preference for light [17]. Therefore, here we address how altering levels of 5-HT transmission in these same ways may affect the strength in the preference for light displayed by individuals. For this, individuals were tested on the dark versus light test (Figure 4A), exposed for 24 h to either 5 uM pCPA (to decrease 5-HT levels) or 1 uM fluoxetine (to enhance 5-HT transmission), then re-tested on the same light versus dark test.

The results show that decreasing 5-HT levels by exposing tadpoles for 24 h to the tryptophan hydroxylase inhibitor pCPA led to a decrease in the strength of the preference for light displayed by the majority of individuals (Figure 4B). Post-pCPA preferences fell by an average of 30% (Figure 4B). We also observed a small subset of tadpoles who appeared unaffected by pCPA exposure. These individuals all displayed an abnormally high preference for light both pre- and post-pCPA exposure (Figure 4B). While this subset is too small for statistical analysis, it does suggest the possibility that these strong preferers of light may not be sensitive to pharmacologically decreased levels of 5-HT.

Conversely, enhancing 5-HT transmission by exposing tadpoles for 24 h to the SSRI fluoxetine increased the strength of the preference for light displayed by the majority of individuals (Figure 4C). Post-fluoxetine preferences increased by an average of 29.3% compared to the pre-fluoxetine score. We also observed that the only tadpoles that did not display an increased preference for light post-fluoxetine were those individuals who displayed the strongest preference for light pre-fluoxetine. This could be interpreted as the innate preference for light being so strong that it occluded the effect of fluoxetine (i.e., the preference for light was essentially maximal, so it could not be increased). The other possibility stemming from our pCPA results is that this subset of tadpoles that display an unusually strong preference for light may not be sensitive to altered levels of 5-HT. We suspect the latter may be the case given that, in general, fluoxetine-induced strengthening of the preference for light did not equal that of the subset of naturally strong preferers.

### 3.3. The Specific Strength of the Preference for Light Displayed by Most Individuals May Be Determined by a Specific Level of Endogenous 5-HT Activity

**Figure 5:**
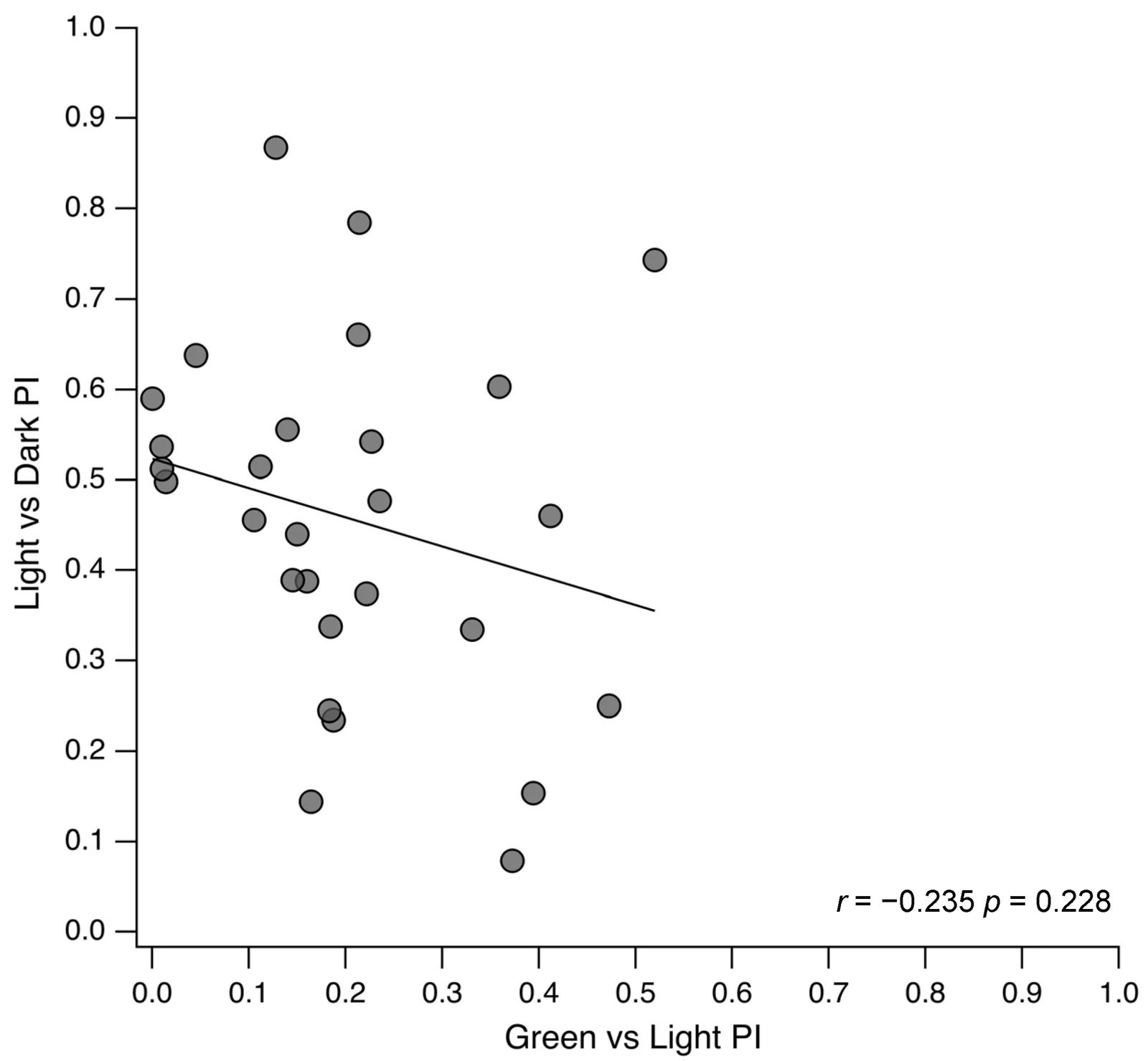
Plotting the preference index (PI) scores for green over light vs. light over dark reveals a weakly negative correlation. Plot showing the preference index values for individual tadpoles for their respective tests (PI score for green vs. light on x-axis, and for light vs. dark on y-axis). The plot displays a weak, statistically insignificant negative correlation between the individual tadpole’s light preference index and their green preference index (r = −0.235 *p* = 0.228; *n* = 28), suggesting that individuals who display a strong preference for light tend to display a relatively weak preference for green and vice versa.

We determined that pharmacologically modulating serotonin activity levels up or down strengthened or weakened, respectively, the preference for light over dark displayed by most individuals. This suggests that the observed variability in innate preferences expressed across tadpoles (Figure 2 and Figure 3) may be due to differing levels of 5-HT activity across individuals. To investigate this possibility, we measured the preference for the color green when green is pitted against light, and for light when light is pitted against dark (i.e., each individual was run on the Green vs. Light, and Light vs. Dark tests, respectively). The logic underlying testing both preferences is as follows: we previously found that increasing 5-HT activity by exposing groups of tadpoles to an SSRI (either trazodone or fluoxetine) strengthened the group’s average preference for light over dark [17], and switched their preference for the color green to a significantly stronger preference for light in the green versus light test [4]. This implies that tadpoles with naturally high levels of endogenous 5-HT activity—like that induced by fluoxetine exposure—would be expected to score especially high on the light versus dark test, and to score especially low on the green versus light test, and conversely individuals with relatively lower levels of endogenous 5-HT activity would be expected to score relatively lower on the light versus dark test and higher on the green versus light test. We plotted each individual’s score on the light versus dark test (y-axis) and their score on the green versus light test (x-axis; Figure 5). While analysis of the best fit line shows that the negative correlation between the two preferences is weak and statistically insignificant, the negative slope is in the direction predicted by our hypothesis, and shows that the preference hierarchy of green > light > dark that was observed for groups of tadpoles does not apply to every individual.

## 4. Discussion

Our previous studies have shown that groups of tadpoles display a preference for green over light and for light over dark [4,17]. Both of these preferences were reported to be moderate in strength and persistent across time. The preferences displayed by the group, however, do not reflect preferences displayed by individuals. There are several ways that this moderate preference could be manifested. For instance, a moderate preference for light over dark could be achieved by (1) all the tadpoles of the group displaying a moderate preference, (2) a bimodal distribution—two subsets, one (slightly larger set) that displays a strong preference and one that displays a weak preference, or (3) a graded pattern of preferences that range from strongly- to weakly preferring individuals. While all of these scenarios would give rise to a moderate group average, they suggest different principles of individual tadpole behavior. Through measuring the preference of single tadpoles, we have determined that individuals do not all display the same moderate preference for light over dark, nor do they display a bimodal pattern. Instead, our data reveal a relatively wide-ranging, graded pattern of preference strengths displayed across individuals and which are consistent within individuals (Figure 2 and Figure 3).

While this suggests the way in which the group average is manifested, we cannot rule out that the behavior displayed by a single tadpole, swimming solo around the test dish, may not be the same as that displayed when the same individual is part of a group. A couple lines of evidence, however, suggest that overall swimming behaviors and preferences of individuals may be similar when tested singly or as a group. First, we previously observed no schooling effect when measuring the preferences of tadpoles in groups of ten [4]. Second, the average of the individual scores reflects a moderate preference for light over dark and for green over light, suggesting that the combination of individual behaviors is how the group average is achieved.

### 4.1. Variation in Visual Preference Across Tadpoles but Consistent Preferences Within Suggests Behavioral Individuality, Aka a “Personality”

The results of this study indicate that tadpoles display enduring preferences within individuals and highly variable preferences across individuals. In animal behavior research, a behavior that displays this within-individual consistency combined with among-individual variability, is referred to as behavioral individuality or personality trait [23,24,25,26,27]. Therefore, the distinct and consistent visual preferences displayed by Xenopus tadpoles suggest bona fide behavioral individuality. While this is, to our knowledge, the first description of behavioral individuality displayed by Xenopus tadpoles, studies on the American Bullfrog tadpole report individuality in exploratory behaviors [24]. And it has been reported that individual wood frog (*Lithobates sylvaticus*) tadpoles display distinct yet consistent levels of exploratory and bold behaviors across different contexts, suggesting behavioral syndromes [28]. Our results may reflect similar personality traits displayed by *Xenopus laevis* because choosing to be in the well-lit area of the test dish is likely an exploratory behavior, and since green is the color of the aquatic plants that provide shelter and food, showing a strong preference for this color may reflect a less exploratory/more timid personality.

### 4.2. Implications of the (Weak) Negative Relationship Between Preference for Green and Preference for Light

Similarly to our previous work on groups of tadpoles, we found that pharmacologically enhancing or decreasing serotonin transmission increased or decreased, respectively, the strength in the preference for light displayed by the majority of individuals that we tested. It follows, therefore, that for most individuals, the specific strength in preference for light may be determined by the individuals’ specific, endogenous level of 5-HT transmission. To further test this possibility, we took advantage of our previous finding that increasing levels of 5-HT transmission by exposing tadpoles to the SSRI trazodone switched their preference from green over light to a much stronger preference for light over green [4]. This previous result suggests that tadpoles with especially high endogenous 5-HT transmission levels should prefer light over green, and those with relatively lower levels should prefer green over light. To determine if this is the case, individual tadpoles were tested on both the light versus dark and green versus light tests. Our data show a negative, albeit weak, correlation between a given individual’s preference index score for light versus dark and green versus light (Figure 5), which is what would be predicted if endogenous 5-HT activity levels were regulating the strength in the displayed preference for light.

### 4.3. Ethological Implications

What may be the benefit of graded pattern of preferences for green and light displayed across tadpoles? One possibility is that a gradient of visual preference would theoretically minimize the amount of overlap of individuals across space which would minimize competing for food in a given area (applicable to filter feeders like tadpoles). Individual visual preferences would also optimize survival of the population—if all displayed the same preferences, then they would tend to form schools, which are easier for predators to detect. Further, this innate gradient would provide the basis for positive feedback loops [27,29]—e.g., if light activates 5-HT production, then those individuals who spend most of their time in the light (potentially due to high level of endogenous 5-HT activity) will activate more 5-HT which will strengthen their preference for light and so forth. This type of positive feedback loop—applied across the gradient of preferences—would theoretically enhance and stabilize the variability expressed across the population.

### 4.4. Limitations

The relatively small sample sizes that comprise this study prevent that conclusion that the few individuals whose exceptionally strong preference for light appeared unaltered by pCPA or fluoxetine exposure are truly insensitive to altered 5-HT activity levels. For this to be determined, larger cohorts will be tested in order to isolate and study this potential subset. Also, our combined results only suggest that differential levels of endogenous 5-HT activity expressed across individuals may underlie the variation in their visual preferences. To directly test this will require measuring 5-HT levels or 5-HT levels of activity, which is beyond the scope of this initial behavioral study. Finally, our analyses were not corrected for multiple comparisons, which renders the results strictly exploratory.

## 5. Conclusions

Overall, our data show that preferences displayed by Xenopus tadpoles are consistent within individuals but variable across individuals, suggesting individuality for these behaviors. We also found that altering 5-HT levels up or down, increased and decreased the preference for light over dark for most of the individuals, and that a small subset that displayed especially strong preferences for light appeared insensitive to these manipulations, suggesting the intriguing possibility that the strength of the preference for light for these individuals may not be 5-HT-dependent. This could reflect a sexual dimorphism. Adult male *Xenopus tropicalis* frogs are known to display exploratory behavior while females display behaviors that range from sedentary to exploratory [30]. Therefore, if choosing to reside in the light region of the test dish is an exploratory behavior, then the subset of individuals that displayed the strongest preference for light and whose preference was not dampened by exposure to pCPA may be male, and the tadpoles whose preferences vary across individuals and appear more 5-HT-dependent may be female. This possibility that the 5-HT-sensitivity may be a sexual dimorphism is consistent with a study in zebrafish showing that exposing female zebrafish to an SSRI amplifies their tendency to be either bold or timid, but has no effect on male behavior [31]. Also, a study in mice shows that stress activates sex-dependent changes in 5-HT receptor expression in a way that renders females more vulnerable to stress [32]. Therefore, future directions include determining whether the sensitivity/insensitivity to altering 5-HT levels is a sexual dimorphism. We will also investigate the effect of social environment on individual visual preferences. Our preliminary findings suggest a potential effect of clutch size on visual preferences. This will be interesting to determine because it is in contrast to the Amazon molly which is reported to maintain behavioral individuality regardless of social environment [23].

## Figures and Tables

**Table 1:** Results of statistical analysis within and across experimental group.

	Test	LvD Day 1	LvD Day 2	GvL Day 1	GvL Day 2	LvD Pre-pCPA Day 1	LvD pCPA Day 2	LvD Pre-FLX Day 1	LvD FLX Day 2
Calculations									
*n*		19		23		12		10	
Standard Deviation		0.20	0.18	0.14	0.15	0.29	0.41	0.32	0.16
CV		51.8%	45.3%	47.3%	52.7%	46.5%	117%	60.86%	23.34%
*p*-value		0.515 ^a^		0.377 ^a^		0.003 ^a^		0.033 ^b^	
ICC, 95% CIs		0.651, (0.295,0.849)		0.717, (0.445, 0.869)					
			

a: Wilcoxon matched pairs signed-rank test; b: Two-tailed paired *T*-test, data fit Gaussian distribution; thus, paired *t*-test was used. Abbreviations: LvD, light vs. dark test; GvL, green vs. light test; FLX, fluoxetine; *n*, number of tadpoles; CV, coefficient of variation; ICC, intraclass correlation coefficient; CI, confidence interval.

## Data Availability

The data presented in this study are available on request from the corresponding author. The GoPro video files are not publicly available in a repository due to their exceptionally large file size.

## Abbreviations

The following abbreviations are used in this manuscript:

5-HT	5-hydroxytryptamine (serotonin)
*X. laevis*	*Xenopus laevis*
ROI	Region of interest
pCPA	4-Chloro-DL-phenylalanine methyl ester hydrochloride
FLX	Fluoxetine, N-methyl-3-phenyl-3-[4-(trifluoromethyl)phenoxy]propan-1-amine
CV	Coefficient of Variation
ICC	Intraclass correlation coefficient
CI	Confidence interval
IQR	Interquartile range
SSRI	Selective serotonin reuptake inhibitor
LvD	Light vs. dark
GvL	Green vs. light
